# Understanding What We See: How We Derive Meaning From Vision

**DOI:** 10.1016/j.tics.2015.08.008

**Published:** 2015-11

**Authors:** Alex Clarke, Lorraine K. Tyler

**Affiliations:** 1 Centre for Speech, Language and the Brain, Department of Psychology, University of Cambridge, Cambridge CB2 3EB, UK

**Keywords:** Concepts, semantics, perirhinal cortex, fusiform gyrus, ventral visual pathway, category

## Abstract

Recognising objects goes beyond vision, and requires models that incorporate different aspects of meaning. Most models focus on superordinate categories (e.g., animals, tools) which do not capture the richness of conceptual knowledge. We argue that object recognition must be seen as a dynamic process of transformation from low-level visual input through categorical organisation to specific conceptual representations. Cognitive models based on large normative datasets are well-suited to capture statistical regularities within and between concepts, providing both category structure and basic-level individuation. We highlight recent research showing how such models capture important properties of the ventral visual pathway. This research demonstrates that significant advances in understanding conceptual representations can be made by shifting the focus from studying superordinate categories to basic-level concepts.

## Trends

We view object recognition as a dynamic process of transformation from low-level visual analyses through superordinate category to basic-level conceptual representations.

Understanding this process is facilitated by using semantic cognitive models that can capture feature-based statistical regularities between concepts, providing both superordinate category and basic-level information.

We highlight research using fMRI, MEG, and neuropsychological and behavioural testing to show how feature-based cognitive models can relate to object semantic representations in the ventral visual pathway.

The posterior fusiform and perirhinal cortex are shown to process complementary aspects of object semantics.

The temporal coordination between these regions is also highlighted, while superordinate category information precedes basic-level semantic information in time.

## Flexible Access to Conceptual Representations

How do we understand what we see? We interpret this fundamental question as asking how visual inputs are transformed into conceptual representations. Our conceptual knowledge (see Glossary) reflects what we know about the world, such as learned facts, and the meanings of both abstract (e.g., freedom) and concrete (e.g., tiger) concepts. Our focus here is on concrete concepts. When conceptual knowledge is accessed, the information retrieved needs to be behaviourally relevant. Acting appropriately requires flexible access to different types of conceptual information. Depending on perceptual context and behavioural goals, objects are recognised in different ways, for example, as a cow, an animal, or living thing. The way objects are naturally recognised is by accessing information specific enough to differentiate them from similar objects (e.g., recognising an object as a cow rather than a horse or a buffalo) – a notion termed the basic or entry-level of representation 1, 2*.* However, part of understanding the meaning of an object also necessitates that more-general information is accessed – for example, the commonalities between similar objects that enable us to know that an object is part of a **superordinate category** (e.g., as an animal or living thing). To understand the cortical underpinnings of this flexible access to different aspects of conceptual representations, we need to specify the neurocomputational processes underlying meaningful object recognition. This in turn requires that conceptual representations are studied as the expression of a set of dynamic processes of transformation – from the visual input and different stages of visual processing in the brain, through different types of categorical organisation, to a basic-level conceptual representation.

Object recognition has generally not been conceptualised in these terms. It is a domain of research that straddles many different subdisciplines – most saliently vision science and semantic memory – but these different strands tend to remain fragmented owing to the complexity and depth of individual areas. A central theme in vision science is to develop computational accounts of the ventral visual pathway, based on visual image properties, which try to explain non-human primate and human brain data (e.g., 3, 4, 5, 6). However, these models are unable to capture the relationships between different concepts – that an apple and a banana are more related than an apple and a ball (which are more visually similar). Further, models of vision alone cannot account for properties such as conceptual priming and flexible access to different aspects of meaning.

Research in semantic memory, by contrast, focuses on the organisation of semantic knowledge in the brain resulting in a variety of accounts drawing upon neuropsychology, functional neuroimaging, computational modelling, and behavioural paradigms. Providing a review of these perspectives is beyond the scope of this article, and many excellent contemporary reviews are available 7, 8, 9, 10, 11, 12, 13, 14. Our focus here is on understanding the neural processes that underpin how meaning is accessed from vision. We describe a neurocognitive model that integrates (i) a cognitive account of meaning based on the statistical regularities between **semantic features** (e.g., ‘has 4 legs’, ‘has a mane’, ‘is black and white’) that can explain a range of semantic effects, with (ii) the neurocomputational properties of the hierarchically organised ventral visual pathway.

## Basic-Level Concepts and their Superordinate Categories

Most cognitive models of object meaning address semantics through one of two approaches – focusing on superordinate category organisation (e.g., 9, 15) or **basic-level concepts** (e.g., [16]). However, a comprehensive account needs to consider both these facets.

**Figure 1:**
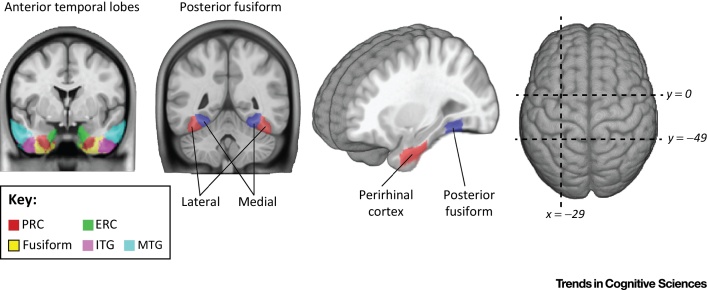
Regions Supporting Conceptual Processing in the Anterior and Posterior Ventral Visual Pathway. Different subregions of the anterior temporal lobe are shown where the middle temporal gyrus (MTG) and inferior temporal gyrus (ITG) are relatively more lateral, the fusiform occupies a ventral position, and the perirhinal (PRC) and entorhinal cortex (ERC) are more medial in the anterior medial temporal cortex (reprinted from [43]).

Research into the organisation of semantic knowledge in the brain has been largely motivated by the observation of semantic deficits resulting from brain damage and disease – most strikingly those deficits that seemed to be specific to only some superordinate categories. Such category-specific deficits after neurological diseases such as herpes simplex viral encephalitis (HSVE) have shown that tissue loss in anteromedial temporal cortex (AMTC; Figure 1) can disproportionately impair knowledge for living things, with relative preservation of knowledge for nonliving things 17, 18. Complementing these neuropsychological data, functional imaging and electrophysiology studies of healthy individuals show increased activity in the AMTC for living things versus nonliving things 19, 20, 21, 22, 23.

**Figure 2:**
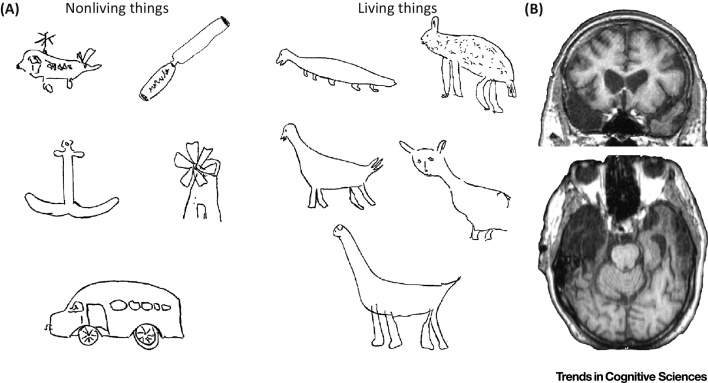
The Nature of Category-Specific Deficits. (A) Drawings from patient SE of common objects of living and nonliving things, showing a clear absence of distinctive feature information for living things and a preservation of details for nonliving things. Nonliving objects, top left to bottom right; helicopter, chisel, anchor, windmill, bus. Living objects; crocodile, zebra, duck, penguin, camel. Reproduced from [17] with permission from Taylor and Francis. (B) MRI scan from patient SE showing extensive damage in the right anterior temporal lobe (ATL; image shown in radiological convention, previously unpublished).

By studying patients showing category-specific deficits following AMTC atrophy, we can gain important insights into the nature of the information that is lost. A striking illustration of this comes from patient drawings, where they are asked to sketch a range of living and non-living objects from memory. In the examples in Figure 2A, all the nonliving objects are well-drawn and easily identifiable, while the drawings of animals mostly reflect their shared properties (e.g., four legs, a tail, eyes, horizontal body), making it impossible to identify them as basic-level concepts. It is clear from these examples that the informational loss underpinning the impairments of such patients involves accessing the distinctive properties of living things, rather than a loss of all information (see [17]). This type of perspective suggests that a more nuanced view of category-specificity in the AMTC is needed, one that takes into account the nature of the deficits at a more specific level than superordinate categories.

Functional brain imaging studies of healthy individuals have provided key evidence that apparent superordinate category effects are not restricted to the AMTC. In the posterior fusiform gyrus (Figure 1), animal images have been shown to produce enhanced effects in the lateral posterior fusiform gyrus, and tool images show effects in the medial posterior fusiform gyrus 15, 24. The nature of this lateral-to-medial gradient in the posterior fusiform is especially intriguing given the range of parameters that produce similar distinctions – such as real world object size [25], animacy [26], expertise [27], and retinotopy [28], suggesting that highly complex representations in this region encompass multiple types of stimulus properties 29, 30.

The effects animals and tools have on the posterior fusiform is one of a range of category-specific effects that have been observed in the temporal and parietal lobes for different categories – animals in the lateral fusiform, superior temporal sulcus (STS), and amygdala 31, 32; tools in medial fusiform, middle temporal gyrus (MTG), inferior parietal lobule (IPL) [33]; places in the lingual, medial fusiform and parahippocampal gyrus [34]; faces in the lateral occipital, lateral fusiform, STS 35, 36; bodies in the lateral fusiform and STS [37]. While understanding the organisation of different categories remains a central issue for cognitive neuroscience, we focus here on one aspect of this, category effects of animals and tools in the posterior fusiform, to illustrate the insights and advances we can make by studying part of this system in detail.

The effects of superordinate category in the AMTC and posterior fusiform must reflect complementary, but different, aspects of semantic computations, but research focusing on superordinate categories has been insufficient to resolve the complementary roles these regions might play.

A largely separate strand of research has focused on basic-level conceptual entities and centres on the anterior temporal lobe (ATL, often defined as the anteroventral and anterolateral aspects of the temporal lobe) which is claimed to represent amodal conceptual information 11, 38. This idea draws upon the notion of convergence zones in the ATL, which acts to bring together information from other brain regions to represent concepts 38, 39, 40. Widespread damage to the ATL is associated with semantic deficits at the level of basic-level concepts for all categories, while superordinate category knowledge itself is unimpaired. Thus, damage to the ATL and to the AMTC seem to have very different effects on **conceptual knowledge** which have yet to be fully explained.

While these lines of research have fundamentally enhanced our understanding of the neural basis of conceptual knowledge, two significant issues arise. First, theories that focus on the organisation of superordinate category information alone ignore what is perhaps the most salient aspect of semantics – the information which differentiates between basic-level concepts – because it is these concepts that are claimed to be the most necessary in daily usage [2]. Consequently, we believe that concepts, not categories, should be the focus of research. Second, research focusing on basic-level concepts has little to say about superordinate category representations. As a consequence, research into superordinate category representations and basic-level concepts is rarely integrated to provide an account of how meaning is accessed from vision.

## Conceptual Structure in the Ventral Visual Pathway

A comprehensive cognitive model of conceptual representations in the brain needs to provide an account of both these sets of issues, and we argue that this can be achieved through the use of semantic feature models of conceptual knowledge. The model that we adopt here, the conceptual structure account 12, 41, claims that concepts can be represented in terms of their semantic features (e.g., ‘has legs’, ‘made of metal’) and statistical measures, termed **conceptual structure statistics**, based on the regularities of features both across concepts and within a concept. Conceptual structure statistics can be informative about both the superordinate category of a concept (e.g., a camel is an animal and a mammal) and how distinctive a concept is within the category (e.g., a camel is distinctive because of its hump which no other animals have). As Box 1 explains, category membership is strongly indicated by the features a concept shares with many other concepts (e.g., many animals have fur, and have legs etc.), while the relationship between the shared and the distinctive features of a concept reflects the ease with which a concept can be differentiated from similar concepts (or conceptual individuation). Further, statistics derived from property norms can reveal systematic differences between categories, such that living things (e.g., animals) have many shared and few distinctive features (all animals have eyes, but few have a hump), whereas nonliving things (e.g., tools) have fewer shared and relatively more distinctive features. The information captured with conceptual structure statistics shows how feature-based models can provide a single theoretical framework that captures information about conceptual representations at different levels of description.

**Figure 3:**
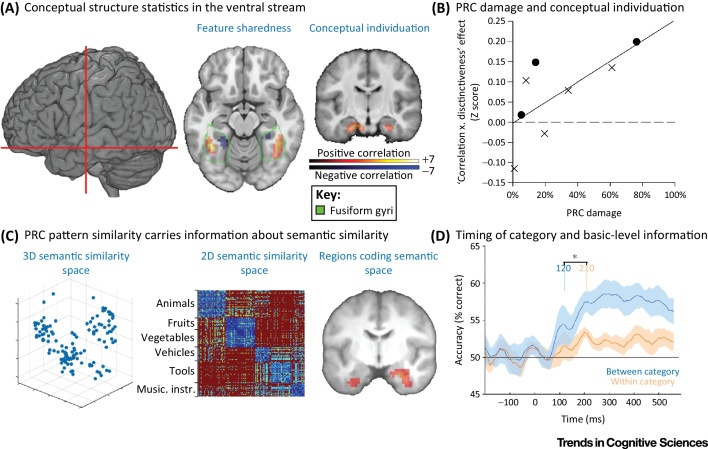
Conceptual Structure Effects In The Ventral Visual Pathway. (A) Conceptual structure statistics modulate activity in both the posterior and anterior-medial temporal lobe based on different feature-based statistics. Posterior fusiform activity increases in the lateral posterior fusiform for objects with relatively more shared features, and activity increases in the medial posterior fusiform for objects with relatively fewer shared features. Bilateral anteromedial temporal cortex (AMTC) activity increases for concepts that are semantically more-confusable (reproduced from [42] with permission from MIT press). (B) Increasing damage to the perirhinal cortex (PRC) results in poorer performance for naming semantically more-confusable objects. This is shown by first correlating the naming accuracy of each patient with a conceptual structure measure for the ease of conceptual individuation. This correlation is then related to the degree of damage to the PRC (crosses denote left hemisphere damage; circles denote right hemisphere damage) (reprinted from [43]). (C) Pattern similarity in bilateral PRC is related to conceptual similarity based on semantic features. Semantic similarity can be defined based on overlapping semantic features between concepts, where concepts both cluster by superordinate category and show within-category variability. Testing the relationship between semantic feature similarity and pattern similarity in the brain shows that bilateral PRC similarity patterns also show a clustering by superordinate category and, crucially, within-category differentiation aligned to conceptual similarity (reprinted from [49] with permission from the Society for Neuroscience). (D) The timecourse of superordinate category and basic-level concept information shown with magnetoencephalography (MEG). Using multiple linear regression we can learn how to map between the recorded MEG data and the visual and semantic measures for different objects. After showing how well this model can explain the observed neural data, we asked how accurately the model could predict MEG data for new objects. This showed than the superordinate category of an object can be successfully predicted before the prediction of the basic-level concept (after accounting for the influence of visual statistics) (reprinted from [68] with permission from Oxford University Press).

Recent fMRI data from healthy participants [42] and lesion behaviour mapping in brain-damaged patients [43] show how conceptual structure statistics – capturing either superordinate category information or the ease of conceptual individuation – differentially relate to regions along the ventral visual pathway. In one study [42], we calculated conceptual structure statistics for a large and diverse set of common objects that participants named during fMRI scanning. We then related brain activation across these objects to different conceptual measures to determine how conceptual structure statistics influence object processing (Figure 3A). The results show that the conceptual structure of an object affects processing at two key sites along the ventral visual pathway. First, there is a gradient effect across the lateral-to-medial posterior fusiform that reflects the mean feature sharedness of a concept. Objects with many shared features (typically animals) show greater effects in the lateral posterior fusiform gyrus, and objects with fewer shared features (typically tools) show greater effects in the medial posterior fusiform gyrus. Second, effects in the AMTC, specifically in perirhinal cortex (PRC), are related to the ease of conceptual individuation: more-confusable concepts evoke greater activation. Evidence from lesion–behaviour mapping [43] confirms this relationship between conceptual structure statistics and the PRC. Damage to the PRC results in an increased deficit for naming semantically more-confusable objects, where confusability is defined by conceptual structure statistics (‘correlation × distinctiveness’; Figure 3B). Together, these two studies converge to highlight a specific relationship, between a conceptual structure statistic capturing conceptual individuation and the PRC, that was only indirectly suggested from prior brain lesion-mapping evidence 44, 45, 46, 47.

The statistical measures derived from feature-based accounts shed new light on the nature of category-specific effects in different regions of the ventral visual pathway, and do so with a framework situated at the level of basic-level concepts. Lateral-to-medial effects in the posterior fusiform gyrus, previously associated with category-specific effects for animals and tools, in fact seem to reflect a gradient of feature sharedness, whereas category-specific effects for living things in the AMTC can be explained in terms of the ease of conceptual individuation – two measures derived from a single account to explain category-specific effects in different regions of the ventral visual pathway for different computational reasons.

This research points to a key computational role for the human PRC in the individuation of semantically-confusable concepts. This role is not relevant for all semantic distinctions, but only for those requiring highly differentiated representations, such as distinctions between a lion, leopard, and cheetah. This is clear from studies showing increased AMTC activity only during basic-level conceptual recognition and not during superordinate category distinctions 22, 48, and from studies showing that activity increases in the PRC during the recognition of semantically more-confusable objects 42, 49.

There are close parallels here with research on the resolution of visual ambiguity and confusability in the PRC in both human and non-human primates 50, 51, 52, and on conceptual effects in humans 23, 42, 46, 49, 53, 54, 55, 56, 57, 58, 59, 60. Functionally, it can be argued that the PRC serves to differentiate between objects that have many overlapping features, and are therefore nearby in semantic space, while objects in sparse areas, with few semantic competitors, require less involvement of the PRC. This is directly supported by research showing that activation patterns in the human PRC reflect the semantic similarity of concepts, as defined by semantic features (Figure 3C) 49, 55.

This computational role of the PRC helps to explain two phenomena from neuropsychology. First, patients who present category-specific deficits for living things following AMTC damage show intact superordinate category knowledge. The basic-level nature of the deficits can be explained in terms of the role of the PRC being predominantly limited to differentiating between entities within superordinate categories. However, not all categories are equally effected following AMTC damage, leading to the second phenomenon: that the observed category-specific deficits for living things occur as a result of a differentiation impairment within denser areas of semantic space, more typical for living things, while these patients can easily differentiate within the less-dense areas typically occupied by nonliving things – resulting in the phenomena seen in Figure 1A.

These findings suggest a conceptual hierarchy in the ventral visual pathway, where a network of regions supports recognition of meaningful objects, and that category-specific effects emerge in different regions owing to categorical differences across complementary semantic feature statistics. This also has the implication that our individual knowledge about objects may reshape the distribution of effects in the ventral stream, consistent with research showing that expertise with different categories, and thus an increased ability to individuate between highly-similar objects, also increasingly engages the lateral posterior fusiform and anterior temporal regions 27, 61 – those regions most important for individuating objects with many shared features and few distinctive features.

## The Temporal Dynamics of Conceptual Processing

We have shown how a semantic feature-based approach can account for observations of superordinate category-specific effects at different loci in the ventral visual pathway. Any comprehensive account of conceptual processing must also be able to capture the temporal dynamics during the retrieval of semantic knowledge. During object recognition, the system dynamics follow an initial feedforward phase of processing as signals propagate along the ventral temporal lobe, followed by recurrent, long-range reverberating interactions between cortical regions 62, 63, 64, 65, 66. The exact nature of the computations supported by these dynamics remains unclear, though there is clear evidence that information relevant to superordinate category distinctions can be accessed very rapidly (within 150 ms 67, 68, 69) whereas specific conceptual information is only accessible after approximately 200 ms 59, 68, 70, 71, 72.

How the temporal dynamics map onto the processing of conceptual information is an issue we have recently begun to investigate [73]. By measuring neural activity with a high temporal resolution, and using machine-learning methods, we can determine whether feature-based models can predict patterns of brain activity over time. One magnetoencephalography (MEG) study along these lines [68] showed that by combining a computational model of visual processing from V1 to posterior temporal cortex [74] with semantic feature information, the neural activity for single objects could be well explained and this model could be used to predict neural activity for other (new) objects. While the model including both visual and semantic information could successfully account for single-object neural activity from 60 ms, the semantic feature information made unique contributions over and above those that the visual information could explain. Semantic feature information explained a significant amount of single object data in the first 150 ms, and this in turn could predict neural activity that dissociated between objects from different superordinate categories. After around 150 ms, the predictions become more specific, and differentiated between members of the same category (i.e., the basic-level concept could be predicted solely based on semantics; Figure 3D).

In a direct assessment of the influence of conceptual structure statistics on the time-course of object recognition, a second MEG study [75] demonstrated that MEG signals correlated with the visual statistics of an object before rapid effects driven by the feature sharedness of the object in the first 150 ms. Subsequent to this, both shared and distinctive features were correlated with MEG signals after 150 ms. Together, these MEG studies highlight two important time-frames of conceptual processing during object recognition – early information that (rapidly activated by visual properties) dissociates superordinate categories and which is driven by shared feature information, and later conceptual integration of information which individuates basic-level concepts from semantically similar items.

## Importance of Anterior–Posterior Interactions in the Ventral Stream

Taken together, data from neuropsychology, fMRI, and MEG reveal that semantic representations are transformed from primarily reflecting superordinate category information to basic-level conceptual information within a few hundred milliseconds, supported by processing along the ventral visual pathway. In particular, the posterior fusiform gyrus and PRC are important to this transition. Electrophysiological recordings in the PRC and posterior ventral temporal cortex of macaques suggest that visual information becomes more differentiated as information flows from posterior to anterior regions [76], a general process along the ventral stream in which object representations are increasingly differentiated [3]. With regards to the mechanism of how basic-level concepts become differentiated within their category, we have shown that connectivity between the ATL and the posterior fusiform increases during tasks requiring access to basic-level concepts compared to those requiring access to superordinate category information [70]. This highlights that the temporal relationship between neural activity in anterior and posterior temporal lobe regions plays an important role in the formation of detailed basic-level conceptual representations.

An important issue is whether interactions involving anterior and posterior regions in the ventral visual pathway are predominantly feedforward or feedback in nature, and how this might change during the course of perception. Combining neuropsychology and functional imaging is particularly illuminating. Patients with semantic deficits following neurological diseases affecting the anterior temporal lobes show reduced functional activity in the posterior aspects of the ventral stream 77, 78, suggesting that anterior damage impacts on the functioning of more-posterior sites. Consistent with this, small lesions to the temporal pole and rhinal cortices (perirhinal and entorhinal) create network dysfunction in the ventral visual pathway, specifically resulting in reduced feedback connectivity from the anterior temporal lobes to posterior fusiform [79]. Overall, these studies strongly suggest that feedback from the anterior temporal lobes, and from PRC, to the posterior ventral stream constitutes a necessary mechanism for accessing specific conceptual representations.

The role that brain connectivity plays in the organisation and orchestration of conceptual knowledge in the brain is yet to be fully appreciated [80]. We have emphasised that connectivity between anterior and posterior temporal lobe sites provides a key underpinning to forming specific basic-level conceptual representations [70], but how this within-temporal-lobe connectivity is coordinated with other networks (e.g., frontotemporal connectivity) remains an important unresolved issue 62, 81. One avenue for progress requires understanding how different brain networks are coordinated, the oscillatory nature of such connectivity and, vitally, how connectivity is modulated by well-characterised and distinct cognitive processes (see Outstanding Questions).

## Concluding Remarks

We have argued here for a single explanatory framework, based on a feature-based account, to understand semantic cognition in the ventral visual pathway. This framework can account for several phenomena, previously unconnected, across behaviour, functional neuroimaging (fMRI, MEG), and brain-damaged patients. Progress in understanding conceptual representations in the brain is significantly advanced by shifting focus to the representation of basic-level concepts and to the relationships between them. We can then harness the potential of large feature-norming datasets to provide well-characterised models of semantic space whose regularities can be exploited using multivariate analysis methods applied to multiple imaging modalities.

Outstanding Questions How does connectivity within, and beyond, the ventral visual pathway emerge and dissolve during the recognition of an object? The way in which regions communicate changes over time, but we know little about how the dynamic patterns of connectivity wax and wane, or what information they reflect.

How does conceptual structure interface with non-visual recognition? The research discussed here is based on visual object recognition, where meaning is accessed from vision. However, it remains to be seen if conceptual structure can account for activations outside the ventral stream, such as during tactile recognition and performing object actions.

How do perceptual and conceptual processes interact during word recognition? Hearing and seeing words will likely have a different conceptual timecourse from viewing images. For a written or spoken word, the form-to-meaning relationship is essentially arbitrary, resulting in different constraints during the transition from form to meaning.

How does expert knowledge influence the dynamics of conceptual processing? It may be the case that becoming an expert for some object classes changes the dynamics of conceptual activation.

What impact does ATL and AMTC damage have on the functional activation of the semantic network? While research suggests widespread ATL damage reduces functional activation in, and connectivity to, the posterior fusiform, the nature of object information we can detect in the compromised network is unknown.

How do concepts come to be represented in the brain the way that they are? Research aiming to uncover what the informational units of meaning are would have a profound effect on theories of semantic cognition.

How is visual information transformed into semantic information? We have shown how different types of perceptual and semantic information can be represented in the brain, although key evidence would be provided by understanding how specific aspects of perception causally activate specific aspects of semantics.

## Box 1 Conceptual Structure Statistics

Many cognitive models of semantics rest on the assumption that meaning is componential in nature, in that a concept is composed of smaller elements of meaning, such as semantic features 12, 82, 83, 84, 85, 86, 87. Semantic features derived from large-scale property norming studies [88] have proven to be a useful way of estimating the underlying structure and content of semantic representations 2, 41, 89, 90. The statistical regularities derived from semantic features, such as the feature frequency and the pattern of feature co-occurrence, correlate with behaviour across a variety of tasks 91, 92, 93 and with measures of brain activity 42, 49, 68, 94, 95, 96, 97, 98.

Research supporting feature-based models highlights three key feature statistics relating to the ease and speed of activating concept-level representations:

First, ‘mean sharedness’ captures whether the semantic features of a concept are relatively more shared by many other concepts (e.g., ‘has ears’) or are more distinctive of the particular concept (e.g., ‘has a hump’). Concepts with many shared features are semantically related to many other concepts, and having many shared features provides a strong indication of superordinate category membership. However, having many shared features also results in increased processing to individuate the concept from their semantic neighbours. A concept that has more distinctive features typically has fewer semantic neighbours, and this facilitates the activation of a unique conceptual representation. Second, ‘correlational strength’ captures how often the features of a concept co-occur and modulate the ease of conceptual processing (the features ‘has eyes’, ‘has ears’, and ‘has legs’ are likely to co-occur with each other). Greater correlation between the features of a concept strengthens the links between them, speeding their coactivation and facilitating conceptual processing. Finally, the interaction between feature sharedness and correlation (‘correlation × distinctiveness’) is thought to play a crucial role in accessing conceptual meaning, such that concepts with highly correlated distinctive features are more easily identified, while concepts that combine highly correlated shared features (such as eyes, ears, legs) and weakly correlated distinctive features (the stripes of a tiger) require additional differentiation processes.

These measures have differential effects depending on the nature of the behavioural goals [93]. During superordinate categorisation (e.g., is an object living or man-made), recognition is facilitated for concepts with many shared features, as well as for concepts whose shared features are more highly correlated. By contrast, during unique conceptual identification (e.g., naming an object as a tiger), recognition is facilitated for concepts with fewer shared features and for concepts whose shared features are more weakly correlated. These contrasting influences of conceptual structure statistics on behaviour reveal how different forms of conceptual information are differentially relevant depending on behavioural goals.

## Glossary

Basic-level concept: we can categorise the same object in many different ways ranging from more to less specific. Examples of the basic-level category are ‘dog’, ‘chair’, ‘hammer’, rather than more-specific (subordinate level; e.g., poodle) or less-specific (superordinate level; e.g., animal) names. The basic-level category of an object is typically the name you would give if asked the question – can you name this object?
Conceptual knowledge: the information we know about things in the world. We use the term conceptual interchangeably with semantic. In contrast to episodic memory, our conceptual knowledge is not tied to any particular place or time; for example, it reflects our knowledge about tigers, rather than our memory of encountering a specific tiger in a specific context.
Conceptual structure statistics: measures based on the regularities and co-occurrences of semantic feature information across different concepts, where the semantic features are typically obtained from large databases (e.g., large norming studies, corpus data). For example, ‘feature sharedness’, or how common a feature is across different concepts, may be calculated as 1/{the number of concepts a specific feature occurs in}. The mean ‘sharedness’ of a concept is then the mean ‘feature sharedness’ over all features in the concept. These statistics can be used to estimate the statistical structure of individual concepts and the relationship of concepts to each other, and have been shown to influence how conceptual information is accessed.
Semantic features: Many models of conceptual knowledge assume that meaning is componential in that the meaning of a concept can be characterised by many smaller units of meaning. Semantic features, such as ‘has legs’ or ‘is round’, are one such approximation of those units and can be derived from property norming studies. Although semantic features are not claimed to be the neural units of meaning, the regularities and statistics derived from them are predicted to share some properties with how meaning is instantiated in the brain.
Superordinate category: refers to groups made up of many concepts, where the grouping is based on semantic properties shared over the group. Superordinate categories can range from more specific categories such as animals, plants, and tools, to less specific categories such as nonliving things (artifacts).
